# Supplementary material for: Solvable constraints and unsolvable limits to global climate adaptation in coastal Indigenous food security
Source: Camb Prism Coast Futur. 2025 Jan 24;3:e3. doi: 10.1017/cft.2025.3 (PMC12337588; doi:10.1017/cft.2025.3)
Supplement: Galappaththi et al. supplementary material [file S2754720525000034sup001.docx]

Table S1: Databases and number of hints per database

| Database | Search string | Hits |
| --- | --- | --- |
| WoS | ((((((TS=((coast*))) AND TS=((communit*) OR (village) OR (rural))) AND TS=((climat*) AND (chang*))) AND TS=((adapt*))) AND TS=((Knowledge))) AND TS=((indigenous) OR (local) OR (traditional))) AND TS=((food*) OR (subsistence) OR (fish*) OR (hunt*)) | [129](https://www-webofscience-com.ezproxy.lib.vt.edu/wos/woscc/summary/d303203f-0c0b-443d-94e4-86defa100779-71c1ff8f/relevance/1) |
| Scopus | ( TITLE-ABS-KEY ( coast* )  AND  TITLE-ABS-KEY ( ( communit* )  OR  ( village )  OR  ( rural ) )  AND  TITLE-ABS-KEY ( ( climat* )  AND  ( chang* )  AND  ( adapt* ) )  AND  TITLE-ABS-KEY ( ( knowledge ) )  AND  TITLE-ABS-KEY ( ( indigenous )  OR  ( local )  OR  ( traditional ) )  AND  TITLE-ABS-KEY ( ( food* )  OR  ( subsistence )  OR  ( fish* )  OR  ( hunt* ) ) ) | [99](https://www.scopus.com/results/results.uri?sort=plf-f&src=s&st1=coast*&st2=%28communit*%29+OR+%28village%29+OR+%28rural%29&searchTerms=%28climat*%29+AND+%28chang*%29+AND+%28adapt*%29%3f%21%22*%24%28knowledge%29%3f%21%22*%24%28indigenous%29+OR+%28local%29+OR+%28traditional%29%3f%21%22*%24%28food*%29+OR+%28subsistence%29+OR+%28fish*%29+OR+%28hunt*%29%3f%21%22*%24&sid=987c6a2912f39fb57b1e614914daf3c1&sot=b&sdt=b&sl=290&s=%28TITLE-ABS-KEY%28coast*%29+AND+TITLE-ABS-KEY%28%28communit*%29+OR+%28village%29+OR+%28rural%29%29+AND+TITLE-ABS-KEY%28%28climat*%29+AND+%28chang*%29+AND+%28adapt*%29%29+AND+TITLE-ABS-KEY%28%28knowledge%29%29+AND+TITLE-ABS-KEY%28%28indigenous%29+OR+%28local%29+OR+%28traditional%29%29+AND+TITLE-ABS-KEY%28%28food*%29+OR+%28subsistence%29+OR+%28fish*%29+OR+%28hunt*%29%29%29&origin=searchbasic&editSaveSearch=&yearFrom=Before+1960&yearTo=Present) |
| Cab Direct | [((coast*)) AND ((communit*) OR (village) OR (rural)) AND ((climat*) AND (chang*) AND (adapt*)) AND ((knowledge)) AND ((indigenous) OR (local) OR (traditional)) AND ((food*) OR (subsistence) OR (fish*) OR (hunt*))](https://www-cabdirect-org.ezproxy.lib.vt.edu/cabdirect/search/?q=((coast*))%20AND%20((communit*)%20OR%20(village)%20OR%20(rural))%20AND%20((climat*)%20AND%20(chang*)%20AND%20(adapt*))%20AND%20((knowledge))%20AND%20((indigenous)%20OR%20(local)%20OR%20(traditional))%20AND%20((food*)%20OR%20(subsistence)%20OR%20(fish*)%20OR%20(hunt*))&sort=Relevance) | [42](https://www-cabdirect-org.ezproxy.lib.vt.edu/cabdirect/search/?q=((coast*))%20AND%20((communit*)%20OR%20(village)%20OR%20(rural))%20AND%20((climat*)%20AND%20(chang*)%20AND%20(adapt*))%20AND%20((knowledge))%20AND%20((indigenous)%20OR%20(local)%20OR%20(traditional))%20AND%20((food*)%20OR%20(subsistence)%20OR%20(fish*)%20OR%20(hunt*))&rowId=1&options1=And&occuring1=All&q1=(coast*)&rowId=2&options2=And&occuring2=All&q2=(communit*)%20OR%20(village)%20OR%20(rural)&rowId=3&options3=And&occuring3=All&q3=(climat*)%20AND%20(chang*)%20AND%20(adapt*)&rowId=4&options4=And&occuring4=All&q4=(knowledge)&rowId=5&options5=And&occuring5=All&q5=(indigenous)%20OR%20(local)%20OR%20(traditional)&rowId=6&options6=And&occuring6=All&q6=(food*)%20OR%20(subsistence)%20OR%20(fish*)%20OR%20(hunt*)&rowcount=6&searchtype=advance-search) |
| AGRICOLA by ProQuest | [((coast*)) AND ((communit*) OR (village) OR (rural)) AND ((climat*) AND (chang*) AND (adapt*)) AND ((knowledge)) AND ((indigenous) OR (local) OR (traditional)) AND ((food*) OR (subsistence) OR (fish*) OR (hunt*))](https://www-cabdirect-org.ezproxy.lib.vt.edu/cabdirect/search/?q=((coast*))%20AND%20((communit*)%20OR%20(village)%20OR%20(rural))%20AND%20((climat*)%20AND%20(chang*)%20AND%20(adapt*))%20AND%20((knowledge))%20AND%20((indigenous)%20OR%20(local)%20OR%20(traditional))%20AND%20((food*)%20OR%20(subsistence)%20OR%20(fish*)%20OR%20(hunt*))&sort=Relevance) | [32](https://www.proquest.com/agricola/results/8663415D57CB4FA1PQ/1?accountid=14826) |

*Table S2: Inclusion and Exclusion Criteria*

| Theme | Guiding Questions | Inclusion | Exclusion | The number of case studies excluded |
| --- | --- | --- | --- | --- |
| Language | Is the paper published in English? | English Only | Non- English | 0 |
| Type of articles | What is the type of the article? | Articles, case studies, workshops, conference proceedings, book chapters, review articles | Editorials, conference abstracts, papers primarily focusing on frameworks or models, and books. | 2 |
| Who adapts | Who adapts? is it about human adaptation | Human | Physical systems, biotic and abiotic systems (e.g., Fish) | 11 |
| Focus | What is the main focus of the publication, is it related to food or food (in)security? | Related to food, food (in) security | Non-related to food, food (in) security, subsistence (e.g., economic valuation of food ) | 2 |
| Time | What is the temporal focus of the paper? | Present | Prehistoric, future/forecasting | 0 |
| Change | Is the paper focus about Climate Change? | Climate Change related | Non-Climate Change related | 0 |

*Table S3: Coding Question*

| 1. What type of limits associated with? | - 1. Hard limits (1=Yes; 0=No)   1.2 Soft limits (1=Yes; 0=No) |
| --- | --- |
| 2. What types of constraints and barriers associated with? (Mark “x” if applicable). | 2.1 Economic (x)  2.2 Social/ Cultural (x)  2.3 Human Capacity (x)  2.4 Governance/ Institutions & Policy (x)  2.5 Financial (x)  2.6 Information/ Awareness and Technology (x)  2.7 Physical (x)  2.8 Biological (x)  2.9 Other (x) |
| 3. Limit thresholds and tipping points | 3.1 Is there any mention of limit thresholds and tipping points? (1=Yes; 0=No)  3.2 Is it a qualitative threshold? (1=Yes; 0=No)  3.3 If yes, add quotes.  3.4 Is it a quantitative threshold? (1=Yes; 0=No)  3.5 If yes, add quotes |
| 4. Severity of the constraints | 4.1 How the severity of the constraints affects the food security in coastal SES? (Open Text)  4.2 Is the paper mention ways of overcoming limits (soft limits)? (1=Yes; 0=No)  4.3 If yes add quotes to support the response |
